# Hybrid metabolic flux analysis: combining stoichiometric and statistical constraints to model the formation of complex recombinant products

**DOI:** 10.1186/1752-0509-5-34

**Published:** 2011-02-25

**Authors:** Nuno Carinhas, Vicente Bernal, Ana P Teixeira, Manuel JT Carrondo, Paula M Alves, Rui Oliveira

**Affiliations:** 1 Instituto de Tecnologia Quimica e Biológica-Universidade Nova de Lisboa/Instituto de Biologia Experimental e Tecnológica (ITQB-UNL/IBET), Apartado 12, P-2781-901 Oeiras, Portugal; 2 Departamento de Bioquímica y Biología Molecular B e Inmunología, Facultad de Química, Universidad de Murcia, E-30100 Murcia, Spain; 3 REQUIMTE, Systems Biology&Engineering Group, Chemistry Department, Universidade Nova de Lisboa, P-2829-516 Caparica, Portugal

## Abstract

**Background:**

Stoichiometric models constitute the basic framework for fluxome quantification in the realm of metabolic engineering. A recurrent bottleneck, however, is the establishment of consistent stoichiometric models for the synthesis of recombinant proteins or viruses. Although optimization algorithms for *in silico *metabolic redesign have been developed in the context of genome-scale stoichiometric models for small molecule production, still rudimentary knowledge of how different cellular levels are regulated and phenotypically expressed prevents their full applicability for complex product optimization.

**Results:**

A hybrid framework is presented combining classical metabolic flux analysis with projection to latent structures to further link estimated metabolic fluxes with measured productivities. We first explore the functional metabolic decomposition of a baculovirus-producing insect cell line from experimental data, highlighting the TCA cycle and mitochondrial respiration as pathways strongly associated with viral replication. To reduce uncertainty in metabolic target identification, a Monte Carlo sampling method was used to select meaningful associations with the target, from which 66% of the estimated fluxome had to be screened out due to weak correlations and/or high estimation errors. The proposed hybrid model was then validated using a subset of preliminary experiments to pinpoint the same determinant pathways, while predicting the productivity of independent cultures.

**Conclusions:**

Overall, the results indicate our hybrid metabolic flux analysis framework is an advantageous tool for metabolic identification and quantification in incomplete or ill-defined metabolic networks. As experimental and computational solutions for constructing comprehensive global cellular models are in development, the contribution of hybrid metabolic flux analysis should constitute a valuable complement to current computational platforms in bridging the metabolic state with improved cell culture performance.

## Background

The biotechnological industry is facing substantial pressure to achieve global process optimization. Concomitantly, the exploitation of high-throughput cellular data to support metabolic engineering has intensified in recent years [1,2]. At the top of the various "omic" layers, the metabolic fluxome resides as their integrated functional output, which in turn closely determines the phenotypic portrait of the cell, particularily productivity [3]. Therefore, the estimation of intracellular fluxes in stoichiometric models is essential [4].

With the emergence of increasingly extensive genome annotations for several organisms, optimization algorithms for genome-wide fluxome estimations have become well popularized. Such estimations are based on flux balance analysis (FBA), which assumes a "metabolic objective" driving the behaviour of the cell (see for instance [5]). A set of derived frameworks have since been developed aiming to probe for genomic alterations (knock-outs, knock-ins, down-regulations and over-expressions) expected to yield better performances than the wild-type [6-9], and examples of successful FBA-driven predictions are available [10,11]. However, because fluxes are estimated in a landscape in which the amount of data is not enough to simply use metabolite balancing, as in small-scale metabolic flux analysis (MFA), the need for additional mathematical assumptions when searching for high yielding genetic alterations may render such predictions less dependable.

In spite of their value, and regardless of size, currently available stoichiometric models by-pass a multi-layered web of regulatory and fundamentally complex cellular events, thus constituting a simplification of cellular function [12]. Given the obvious gap between genome and fluxome, mutants "created" *in silico *by linear optimization/inspection may not exhibit the expected metabolic behaviour *in vivo*. While methods for integration of regulatory and metabolic networks have been reported [13-15], still fragmentary knowledge of kinetic and regulatory phenomena (e.g. transcriptional, translational, signalling), together with their cumbersome biological specificity [16], may preclude their full applicability in metabolic engineering. This is especially relevant when attempting to engineer complex, multi-genic phenotypes, such as improving the yields of recombinant proteins derived from animal cell cultures, for which reason stoichiometric models have been mostly confined to microbial systems producing small molecules [5]. In this respect, an additional important pitfall is the lack of mechanistic knowledge of processing pathways associated with the formation of proteins or viruses; simply lumping the necessary precursors in a set of synthesis reactions has proven a fruitless task since the stoichiometric requirements for these recombinant products are several orders of magnitude below those for host biomass formation, thus making their distinction practically impossible [17,18]. As a result, the application of stoichiometric models to complex product formation has been restricted to the evaluation of optimal conditions for cell growth and metabolic efficiency rather than explicitly defining productivity in the model [19,20].

These issues are herein addressed by proposing a hybrid stoichiometric/statistical framework to make sense of fluxome data either from small-scale or genome-scale metabolic models. As intended for rapid bioprocess optimization, a scenario is presented where MFA is employed to reliably estimate intracellular flux distributions in a small-scale network comprising the main pathways of carbon and nitrogen flow, substantially reducing data acquisition efforts in the early stages. Then, projection to latent structures (PLS) [21] is used to search for correlations between a measured productivity target and the estimated metabolic state, whereby the inclusion of a statistical model allows filling the gaps in our knowledge of global regulation of anabolic processes.

In order to illustrate this approach, the metabolic behaviour of the baculovirus-insect cells system is explored. The superior versatility and safety of baculoviruses has been exploited for a wide range of applications, from recombinant protein manufacture [22,23] to gene expression in mammalian cells, including pharmaceutical screening and *in vivo *gene therapy [24]. Our group has developed rational strategies for baculovirus production optimization in insect cell cultures. These works were based on classical MFA [25,26], from which hypotheses on how to impact metabolism towards a higher productive state were tested and analyzed [27]. Here, these results are combined with new data to demonstrate the ability of hybrid MFA to assign individual fluxes/pathways of central metabolism to cell-specific functions that cannot be completely defined in a stoichiometric description.

## Results

### 1. Limitations of classical metabolic flux analysis

**Figure 1:**
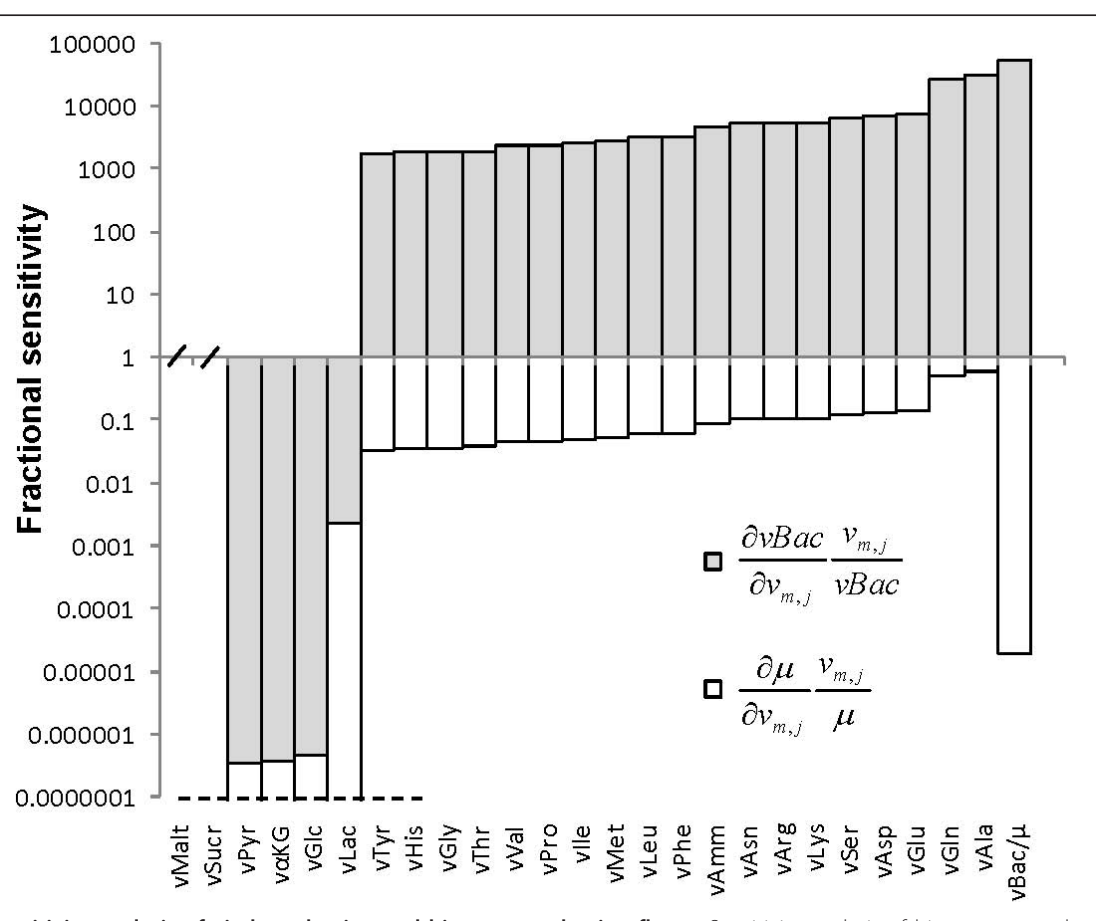
**Sensitivity analysis of viral production and biomass production fluxes**. Sensitivity analysis of biomass or product synthesis estimations was performed when the measured value of each of these fluxes was individually omitted from the complete model, yielding an overdetermined system of equations (see text and Methods section). The estimation of fractional sensitivities was achieved by factoring the appropriate average value of measured metabolite consumption/production rates and average value of measured cell growth rate or productivity, respectively, from all available culture conditions (see Table 2). Horizontal axis marks indicate computed sensitivities are zero.

Supposing the composition of a recombinant product of interest is known (e.g. protein amino acids sequence or detailed virus structure), a lumped reaction system comprising all metabolic precursors in stoichiometric quantities to synthesize a unit amount of product can be formulated. If the number of measured flux constraints is high enough to yield a determined or overdetermined system, then an estimation of the rate of product synthesis can be obtained (see Methods for a description of MFA). However, this estimation will generally be inaccurate, often lying outside feasible biological boundaries, even for a consistent, overdetermined system with carbon and nitrogen balances closed within experimental error. The problem of the ill-definition of protein/virus formation in a metabolic network is mathematically expressed by the extremely large sensitivity of such estimation to the measured fluxes. As a corollary, it is only possible to accurately estimate such a rate if all measurements are precisely known, that is, devoid of experimental error. To illustrate this, a comprehensive metabolic model of *Spodoptera frugiperda *cells (*Sf*9) comprising a well-defined central carbon and nitrogen metabolism (see Additional file 1: Metabolic reactions of the *Sf*9 cell line metabolism) was combined with a set of biosynthesis reactions for recombinant baculovirus synthesis (see Additional file 2: Viral synthesis reactions used for complete MFA model establishment). This network contains 51 independent material balances (54 balanced metabolites minus 3 independent conservation relations corresponding to NADH + NAD^+ ^= a, NADPH + NADP^+ ^= b and FADH_2 _+ FAD = c) and 77 fluxes, resulting in 26 degrees of freedom. Since a total of 30 fluxes are measured or defined, the final system is overdetermined with 4 redundant measurements. Then, the relative sensitivity of the rate of product formation in this metabolic network was compared to that of the cell growth rate (see Methods). As presented in Figure 1, the existence of measurement errors is significantly dampened when estimating the rate of biomass formation (*μ*), having sensitivity values lower than 1 (calculated as the fractional variation in *μ *produced by an infinitesimal fractional variation in a given measured flux). However, they are enormously amplified when estimating the flux of baculovirus formation (*vBac*), often by several orders of magnitude.

If a representative scenario is chosen, the impact of individually omitting the measurements of *μ *or *vBac*, or of deleting the complete set of biomass or product synthesis reactions, can be assessed in terms of overall fluxome variation in relation to that estimated by the complete model (Table 1). As shown, leaving out *μ *has a small effect in the fluxome estimation; moreover, the respective estimated *μ *corresponds roughly to the measurement in the complete model, indicating that the assumed stoichiometry is consistent with experimental data. On the other hand, leaving out *vBac *has a more profound effect in the intracellular flux distribution, evidenced by the considerably higher total fluxome variation. As expected, the estimated viral replication rate is completely unrealistic, even though the model remained consistent, evidencing that viral replication cannot be directly inferred from the nutrients uptake and metabolites production rates affected by experimental error. This is a result of the negligible requirement of anabolic precursors for viral synthesis as compared to biomass formation. Therefore, it is essentially impossible to accurately estimate how much complex product is being synthesized, per cell and unit time, for a given metabolic state. Finally, taking out reactions associated with virus biosynthesis has a virtually null effect on the remaining fluxes, which contrasts with the massive fluxome variation obtained after deleting reactions for biomass formation. It is important to note that model consistency is independent of incorporating the stoichiometry of virus formation. This clearly shows that the mechanisms of complex product synthesis are ill-defined in a purely stoichiometric description.

**Table 1:** Impact of biomass and virus synthesis information on the estimation of *Spodoptera frugiperda*'s post-infection metabolism

	Complete model	W/o *μ*	W/o *vBac*	W/o biomass reactions	W/o viral reactions
Fluxome var. ^a^	-	12.2	32.5	370.2	0.0
*μ *^b^	8.6	8.9	8.0	-	8.6
*vBac *^c^	920.5	920.5	3995861.2	920.6	-
Redundancies ^*d*^	4	3	3	4	4
*h/χ^2 e^*	0.3/7.8	0.0/7.8	0.0/7.8	102.0/9.5	0.3/9.5

^a^Fluxome variation (%), calculated as the average relative variation of all intracellular fluxes estimated by the model, excluding biosynthetic reactions for biomass or virus formation.

^b^Adjusted (underlined) or estimated specific biomass formation rate (h^-1 ^× 1000).

^c^Adjusted (underlined) or estimated specific viral synthesis rate (10^3 ^infectious particles × (10^6 ^cells × h)^-1^).

^d^The number of redundancies in an overdetermined model corresponds to flux measurements in excess of the degrees of freedom of the system (see Methods).

^e^A consistency index (*h*) that is lower than the corresponding *χ^2 ^*(95%) value indicates consistency between flux measurements and the assumed network stoichiometry in each case (see Methods).

### 2. Hybrid metabolic flux analysis

**Figure 2:**
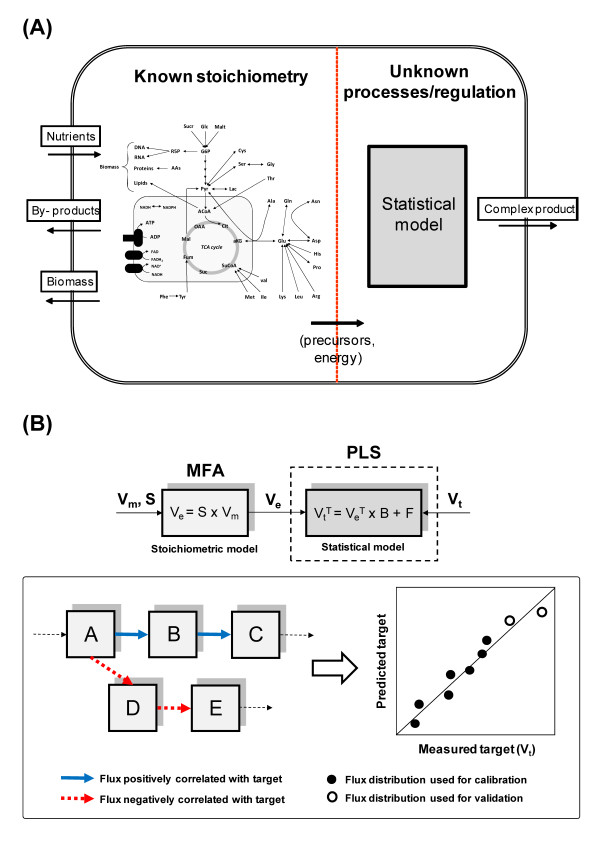
**Data-driven framework for predictive metabolic flux analysis**. (A) Schematic representation of a metabolic network with an unknown or ill-defined portion corresponding to the synthesis of a complex recombinant product. These poorly defined pathways are substituted by a statistical sub-model bridging the known well-defined stoichiometry with the target product formation rate. (B) Given a set of measured fluxes (**V_m _**- usually exchange fluxes of metabolic consumption and production), metabolic flux analysis is used to estimate the entire flux distribution (**V_e_**) in a predefined metabolic network. Then, PLS is performed to find a linear regression model between the estimated fluxome and the vector of a measured target such as productivity, **V_t_**. As a result, a list of regression coefficients representing how strongly each flux correlates with the target is obtained (**B**), making it possible to predict the productivity of independent cultures after metabolic manipulation.

In the absence of sufficient mechanistic detail, the unknown or ill-defined part of a metabolic network can be substituted by an empirical (statistical) sub-model bridging the well-defined stoichiometry with a given cellular function, such as the synthesis of a complex recombinant product (Figure 2 (A)). The herein proposed hybrid MFA takes advantage of using PLS to find a regression model between input fluxes, **V_e _**(estimated through classical MFA), and a vector of target productivity, **V_t_**, which is not directly linked with the known part of the network (Figure 2 (B)). The result is a vector of regression coefficients (**B**) representing how strongly each flux correlates with the target. This statistical association can be used to derive hypothesis on how to perturb the metabolic network towards increased productivity (refer to Methods for a detailed mathematical description).

To assess the performance of this methodology, original and published experimental data were pooled together, comprising a diverse set of 13 independent infection cultures (Table 2). Included are infections performed with a recombinant baculovirus, at different cell densities, in different culture systems and subject to various supplementation and treatment schemes (see details in Methods). In all experiments, the *Sf*9 insect cell line was infected at a low multiplicity of infection (number of infectious particles added per cell) in serum-free medium. Overall, a matrix of 13 × 47 fluxes estimated by MFA was used as predictor data for the PLS model (Additional file 3: Values of measured and MFA-estimated fluxes for all experiments). In all cases, the pseudo-steady state hypothesis on intracellular metabolites was assumed, since extracellular metabolite profiles were approximately linear during the initial 48h to 72h productive phase after infection. Data from the end of infection cultures were not taken into account to avoid confounding effects due to cell lysis. The number of measured fluxes was in excess of the degrees of freedom of the system, resulting in an overdetermined model with 4 redundant measurements. On average, nitrogen and carbon balances closed to 70% and 88%, respectively, and experimental data were consistent with the assumed biochemistry and the pseudo-steady state hypothesis (see Methods). As target, specific baculovirus productivities were measured as described in Methods, covering on the whole 3 orders of magnitude.

**Table 2:** Experimental cultures used for model establishment

#	Virus	CCI^a^	Culture system	Supplementation/Treatment^b^	Productivity^c^	References
1		1		-	1420.7	
2		3		-	117.0	
3		3		Gln	174.3	
4		3		Amino acids mix	177.1	
5	*Ac-VP39EGFP*	3	Spinner vessel	IMS	227.3	[27]
6		3		Pyr	644.2	
7		3		α-KG	920.5	
8		3		Pyr/α-KG	462.4	
9		3		Pyr (24 mM)	455.2	
	
10				-	23.2	
11	*Ac-VP39EGFP*	3	Shake flask	-	29.1	This work
12				PBS (50%)	0.9	
13				AICAR	3.1	

In every case, cells were grown to the appropriate cell density in SF900II serum-free medium and infected with 0.1 infectious particles per cell.

^a^Cell concentration at infection (10^6 ^cells × mL^-1^); "1" and "3" stand for 1-1.5 and 3-4 × 10^6 ^cells × mL^-1^, respectively.

^b^Nutrients supplementation and culture treatment were performed as described in Methods (see indicated references for details). IMS - complete insect medium supplement.

^c^Measured specific viral synthesis rate (10^3 ^infectious particles × (10^6 ^cells × h)^-1^).

**Figure 3:**
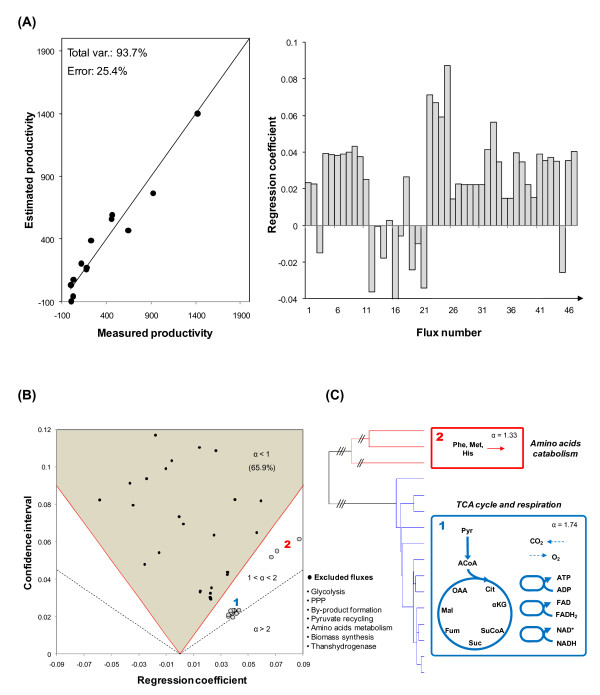
**Functional metabolic decomposition of baculovirus-producing *S. frugiperda*'s cells**. (A) Performance of predictive MFA in describing baculovirus productivity based on metabolic data from 13 independent cultures (see Table 2). In all cases, *Sf*9 insect cells were infected with a low multiplicity of infection in serum-free medium. A total of 3 latent variables were used to describe the data. Productivity is expressed in 10^3 ^infectious particles × (10^6 ^cells × h)^-1^. (B) In order to estimate confidence intervals for the model regression coefficients, Monte Carlo sampling was used to generate 1000 data matrices based on the error variances of all predictor and target variables (see Methods section for details). The strength of association (*α*) was defined as the confidence interval to regression coefficient ratio, allowing the exclusion of those fluxes with *α *values lower than 1. (C) After hierarchical clustering, the TCA cycle and mitochondrial respiration naturally arised as a closely connected group of fluxes strongly correlated with high productivities. With lighter association strengths, one additional cluster corresponds to the catabolization fluxes of the essential amino acids phenylalanine, methionine and histidine. Abbreviations: ACoA (acetyl-coenzyme A), Cit (citrate), Fum (fumarate), His (histidine), Mal (malate), Met (methionine), OAA (oxaloacetate), Phe (phenylalanine), Pyr (pyruvate), Suc (succinate), SuCoA (succinyl-coenzyme A), αKG (α-ketoglutarate).

Our PLS model was able to capture most of the variance in the target productivity, despite using only 3 latent variables to describe the input data (Figure 3(A)). Because in latent variable models, such as PLS, there is no direct relationship between predictor and target variables, the calculation of reliable confidence intervals for the regression coefficients is not straightforward, limiting their interpretability. Namely, regression coefficients in PLS models have been found to reflect primarily the underlying latent structure of the data, rather than accounting for the error variances of the predictor variables [28]. To circumvent this problem, Monte Carlo sampling was used to generate 1000 data matrices based on the error variances of all 47 metabolic fluxes and specific baculovirus productivity. PLS models were then built on the generated data, allowing the estimation of confidence intervals associated with each regression coefficient. The procedure revealed that 31 out of 47 regression coefficients were not statistically meaningful, having strengths of association (*α *- here defined as the ratio between the regression coefficient and the respective confidence interval) lower than 1 (Figure 3(B)). It should be underlined that the flux discrimination criterion includes both observed correlations with the target and precision of measurement/estimation.

The reduced list of meaningful fluxes was then hierarchically clustered in the regression coefficient/confidence interval space in order to highlight groups that share a common strength of association with the target (Figure 3(B),(C)). Overall, the generated cluster tree preserved distance measurements between pairs of data objects, having a cophenetic correlation coefficient of 0.97. To evaluate natural divisions in the dataset, a preliminary analysis was performed by calculating an inconsistency coefficient for each link in the cluster tree (see Methods). Essentially, two clusters stand out, one having *α *values close to 2 corresponding to the tricarboxylic acids (TCA) cycle and respiration reactions, and another, with lighter association strengths, comprising the uptake fluxes of phenylalanine, methionine and histidine. Remarkably, this selected group of amino acids sharing a positive correlation with productivity had on average higher ratios of catabolization (relative to their specific consumption) than the rest of the essential amino acids, which were primarily used for biomass synthesis; the interpretation of these selections can in principle be based on their contribution to oxidative metabolism as well.

In view of these results, engineering strategies aiming to increase carbon flow through central oxidative pathways, possibly by rearranging flux partitioning at key metabolic nodes or feeding energy-generating metabolites, could potentially be beneficial for virus replication. To this respect, it is possible to find in Table 2 a group of cultures that, based on previous empirical inspection, were purposefully designed to increase productivity by the addition of energy-generating metabolites (experiments 6, 7, 8, 9) [27]. Metabolic node rearrangement was not an issue since *Sf*9 cells naturally possess a highly efficient oxidative metabolism [25,27]. Additionally, in face of preliminary simulations, we thought of some treatment to simulate a depressed energetic state and negatively impact productivity, namely through the addition of AICAR (aminoimidazole carboxamide ribonucleotide, experiment 13), a cell permeable AMP mimetic that strongly binds AMP-activated protein kinase, inducing downstream effects typical of ATP starvation such as inhibition of protein synthesis [29]. This experiment, together with the substitution of half the culture medium by PBS (experiment 12), comprised the lower segment in the baculovirus productivity range.

### 3. Predictive power

**Figure 4:**
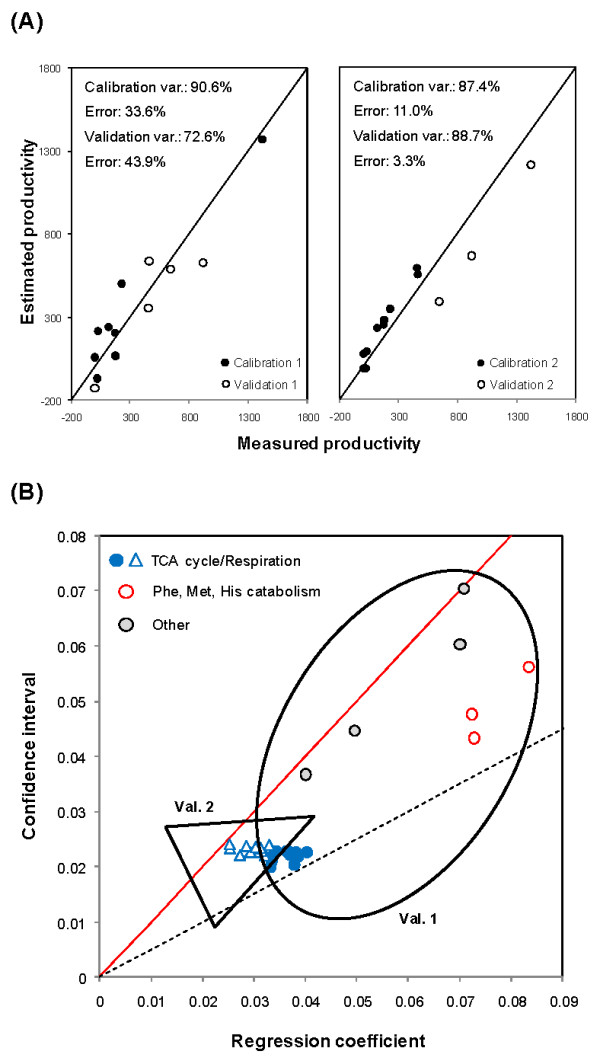
**Validation of predictive MFA for metabolic engineering**. (A) To validate our framework as a powerful tool to assign targets for metabolic engineering, the complete set of 13 experiments was purposefully split into calibration and validation subsets in two manners. In strategy 1, experiments 6, 7, 8, 9 and 13, which have been rationally designed to manipulate the cellular energetic state, were left aside for validation. In strategy 2, the top three producers (experiments 1, 6 and 7) were chosen as validation cultures to avoid data interpolation. The number of latent variables used to build the calibration models in each case was 1 and 3, respectively. Productivity is expressed in 10^3 ^infectious particles × (10^6 ^cells × h)^-1^. (B) Metabolic decomposition for each validation strategy, showing the common selection of TCA cycle/respiration as important pathways for viral replication. Also for validation strategy 1, the catabolism of phenylalanine, methionine and histidine had *α *values higher than 1, as well as other fluxes with lighter correlations with the target (other: catabolism of maltose, proline and tyrosine, formation of alanine).

To validate an empirical model it is mandatory to test its predictive power with new independent experiments not used in the calibration step. We selected the above mentioned subset of experiments (6, 7, 8, 9 and 13) to be used for validation. In a second validation strategy, the three experiments with the highest productivities (1, 6 and 7) were left aside as a means to prevent data interpolation. In both strategies, the number of latent variables was chosen based on maximum total variance explained (calibration and validation) and constrained by a limit of 3. As presented in Figure 4(A), the productivities of experiments 6, 7, 8, 9 and 13 were reasonably predicted, considering the challenging partition of the data (validation 1), while the results for validation 2 were considerably sounder. More importantly, the hybrid MFA-PLS structure clearly outperformed classical MFA in modeling baculovirus production, as demonstrated by comparing the predicted productivities obtained in both validation data sets with the corresponding values predicted by MFA (Table 3).

**Table 3:** Predictive power of the hybrid MFA-PLS structure compared to MFA

Experiment #	Productivity^a^	MFA prediction	Hybrid MFA prediction
1	1420.7	28915535.7	1214.6^c^
6	644.2	-15477747.1	389.9^c^/586.4^b^
7	920.5	3995861.2	665.7^c^/625.6^b^
8	462.4	18837398.3	637.7^b^
9	455.2	9971383.5	353.5^b^
13	3.1	-7199746.4	-130.8^b^

^a^Measured specific viral synthesis rate (10^3 ^infectious particles × (10^6 ^cells × h)^-1^).

^b^Validation strategy 1.

^c^Validation strategy 2.

In terms of metabolic decomposition, these models corroborate our previous conclusions. Indeed, the most important pathways for viral replication previously discriminated, namely TCA cycle and respiration, showed a significant correlation with productivity for both validation strategies (Figure 4(B)). Also for validation strategy 1, the catabolism of phenylalanine, methionine and histidine had again *α *values higher than 1, though other fluxes with lighter correlations were also selected. Overall, despite the somewhat limited collection of data, these results indicate the hybrid MFA framework should prove a valuable tool in designing metabolic optimization strategies for complex products, with potential applicability to a range of cellular systems.

## Discussion

In this work, a cost-effective hybrid methodology is reported to make sense of accessible fluxome data for rapid optimization of complex productivity phenotypes. PLS modelling is used in tandem with classical metabolic flux analysis to establish a link between an estimated metabolic state and system productivity, therefore providing a predictive *in silico *platform to assist genetic/environmental metabolic engineering when a well-defined stoichiometric description of product formation is not available. An important feature of PLS is that it decomposes complex data sets into subsets of uncorrelated vectors, called latent variables, while eliminating redundant information. This permits to address biological problems where the number of variables assessed largely exceeds the number of observations, reason why this method has gathered significance in interpreting "omic" data sets [30]. As reviewed in Teixeira et al. [31], combining such data-mining tools with mechanistic models gives rise to hybrid parametric-nonparametric systems, which enable cost-effective analysis of complex problems with fragmentary knowledge.

Our method is conceived to perform on the basis of an informative, yet not exhaustive, preliminary set of experiments easily available at laboratory scale. It is especially suited to deal with complex products whose synthesis mechanisms are ill-defined by considering a simple stoichiometric description as part of a global metabolic model, or whose composition is unknown. Productivity enhancement in the case of simpler molecules, for instance amino acids or TCA cycle intermediaries, has been previously achieved by stoichiometric analysis of their respective synthesis pathways [32,33]. Here, the main output is the global identification of fluxes strongly correlated with a highly productive state, which are discriminated from a background of less significant metabolic reactions contributing to product synthesis. Thus, as opposed to classical MFA, our approach enables predicting the productivity in independent experiments based on a previous calibration, and the identification of metabolic targets for production optimization. In this respect, the estimation of reliable confidence intervals for the flux regression coefficients is crucial to remove a large portion of uncertainty in the selection of metabolic targets, considerably improving the odds of successful experimental validation. Methods that assure a higher precision in fluxome estimations, such as isotopic tracer experiments [34], could in principle expose other targets for manipulation.

It should be noted that the predictive capacity for the phenotypic change does not necessarily translate in the ability to predict the means to deliver this change. Specifically, finding a strong statistical correlation between a given metabolic route and productivity does not translate into a direct cause-effect relationship. While this may often be the case for the synthesis of single molecules, production of correctly formed proteins or viruses depends on a complex series of steps ranging from gene transcription to protein secretion or virus assembly, along with their regulation through even less understood signalling events (35). Therefore, the identification of genetic targets may at times be beyond the domain of central metabolic fluxes, which themselves are upstream regulated along with the productivity phenotype. On this issue, the methodology herein presented could at least allow to hypothesize how different cell pathways/functions are commonly regulated.

Besides providing a list of prospective metabolic targets to be exploited for engineering, the proposed framework adds a functional dimension to previous metabolic decomposition studies based solely on structural properties of the underlying network, namely connectivity [36,37] or pathway feasibility [38,39]. Here, clusters of fluxes are defined by sharing the same relationship with a given cellular output. As a main drawback, our method is constrained by data availability on cellular fluxome and target phenotype, thus demanding some experimental effort.

As mentioned earlier, when used to handle flux distributions estimated by FBA in genome-scale models, this approach represents an alternative hybrid framework to linear optimization techniques during metabolic target selection, which could significantly surpass existing limitations in modeling complex phenotypes. A recent paper by Melzer et al. (2009) also explores the use of multivariate statistics to correlate stoichiometrically-derived elementary modes in complex networks with stoichiometrically-defined productivity targets, as opposed to common search algorithms for strain improvement [40]. However, our approach differs conceptually to the cited work in that we define a statistical bridge between a well-defined stoichiometry and a complex phenotype, therefore substituting for a metabolic link that may be ill-defined in a purely stoichiometric representation and otherwise hampered by insufficient kinetic and regulatory information. This should prove an advantage in predicting non-obvious metabolic targets associated with the synthesis of more complex recombinant products in animal cells, particularly multimeric proteins, multi-protein particles and viruses, the later adding an additional degree of complexity due to the virus-coded regulation of cellular machinery.

Finally, from a practical point of view, several issues are worth considering before opting for a genome-scale model. In one respect, the availability of a well sequenced and annotated genome may constitute a major limitation: while accurate metabolic reconstructions are available for un-mutated, standard microorganisms such as *E. coli *and *S. cerevisiae*, in an industrial setting a larger diversity of organisms are used, particularly animal cell systems for which cellular data is much scarcer [41]. Another consideration is the cost and time associated with the creation of these models. Even if a comprehensive genome-scale stoichiometric model is already at disposal, a considerable experimental effort is necessary to overlay high-throughput metabolomic and isotopomer flux data for better constraining fluxome estimations [42]. In particular, the computational power required for ^13^C flux analysis may become prohibitive for very complex networks by today's standards. Overall, our framework could potentially be more useful to steer rapid development of a broad range of organisms on the basis of a representative small-scale metabolic network. As such, it would significantly enhance the quality of information extracted from exploratory experiments compared to traditional metabolic flux analysis.

## Conclusions

The need to understand and manipulate cellular systems for increased biosynthesis of target products has been a consistent focus of research, now supported by the huge flows of data being obtained at different cellular levels. However, the increasingly available knowledge is still not sufficient for the construction of global metabolic models able to accurately predict cell behaviour in response to perturbations. To this end, multivariate statistical tools will keep proving useful in functionally connecting different layers of cellular information, filling the gaps in our understanding of kinetic and regulatory phenomena. At this point, we believe that combining both frameworks into a hybrid metabolic flux analysis framework constitutes a valuable and straightforward complement to purely stoichiometric models in optimizing industrially relevant complex productivity phenotypes.

## Methods

### Metabolic flux analysis

In MFA, material balances are derived over the metabolic nodes of a biochemical network. Under the pseudo-steady state assumption, all intracellular pools of these compounds are constant, and a homogenous system of linear equations is obtained

(1) 0=A×V,

with **A **the network stoichiometric matrix and **V **a vector of reaction fluxes. When enough constraints are available to render system (1) overdetermined (that is, determined and redundant with flux measurements in excess of the degrees of freedom of the system), a least squares solution for the flux distribution can be derived:

(2) $$V_{e}=-A_{e}^{\text{\#}}\times A_{m}\times V_{m},$$

with **V_m _**a vector of measured fluxes (usually consumption/production rates of metabolites and cell growth), **A_m _**the associated stoichiometric matrix, **V_e _**the vector of estimated intracellular reaction rates and **A_e_^# ^**the pseudo-inverse of the corresponding stoichiometric matrix. To provide an estimate that better approximates the real metabolic flux distribution, redundant measurements were used to balance (adjust) the measured fluxes according to their normalized variances to obtain a weighted-least squares solution [43]. In addition, adjusted flux measurements were used to calculate a consistency index, *h*, as defined in [44]. Comparison of *h *with the χ^2 ^test function allows evaluating the consistency of experimental data with the assumed biochemistry and the pseudo-steady state assumption. For this, the number of redundant measurements was used as the degrees of freedom for statistical hypothesis testing at a 95% confidence level. These calculations were performed with FluxAnalyzer (Version 5.3) [45].

### Sensitivity analysis

For the two scenarios where the measurement of product formation rate or cell growth rate are individually omitted from the complete model, the Jacobian of the corresponding overdetermined system (2) was calculated as

(3) $$J_{V_{e}\left(V_{m}\right)}=-A_{e}^{\text{\#}}\times A_{m}={\langle \frac{\partial v_{e,i}}{\partial v_{m,j}}\rangle }_{i,j},$$

representing the absolute sensitivity of each unknown flux to each metabolic constraint. In order to compute fractional sensitivities for biomass and product synthesis rates, the corresponding (i,j) elements were appropriately factored with an average value of each measured metabolite consumption/production rate and the average value of measured cell growth rate or productivity, respectively, for all metabolic conditions presented in Table 2.

### Hybrid metabolic flux analysis and Monte Carlo sampling

After standardizing the fluxes estimated by MFA, the decomposition of the predictor matrix **V_e_^T ^**(with different culture conditions/steady states as rows) is performed iteratively and supervised by maximizing covariance with the target vector **V_t_^T^**. The result is the projection of the fluxome space into a "latent space" defined by a set of uncorrelated (orthogonal) latent variables (LVs):

(4) $$T=V_{e}^{T}\times W,$$

(5) $$V_{e}^{T}=T\times Q^{T}+E,$$

where **T **is the matrix with LVs as columns, **W **is a matrix of weights, **Q^T ^**a matrix of "loadings", and **E **a residuals matrix. Similarly, the target vector can also be decomposed using the same latent space as

(6) $$V_{t}^{T}=T\times P^{T}+F,$$

with **P^T ^**the corresponding loadings vector and **F **a vector of residuals. Finally, the linear model between the predictor and target variables is derived:

(7) $$V_{t}^{T}=V_{e}^{T}\times B+F.$$

Here, **B **= **W **× **P^T ^**is the column vector that maximizes the squared sample covariance between **V_t_^T ^**and the latent variables in **T **[30]. It represents the set of regression coefficients establishing the statistical relationship between fluxome state and the target cellular function, containing quantitative and qualitative information on the potential impact that each flux has on the target variable. The main algorithm for these calculations is part of "The *N*-Way toolbox for MATLAB" [46] and is described in [47].

For the estimation of confidence intervals associated with each regression coefficient, 1000 fluxome states were randomly sampled considering normal error distributions associated with flux estimates that have been propagated in MFA calculations from an initial 16% error in measured exchange fluxes (including cellular growth rate). For the productivity target, a 22% error was used [48]. PLS regression was then performed for the complete generated set of data, resulting in a 1001-sized population of regression coefficients for each flux. Confidence intervals were computed as

(8) $$B\pm \overset{\wedge }{\sigma }\text{(}B\text{)}\times T_{(0.975,\mathrm{dg})},$$

with $\overset{\wedge }{\sigma }$ (**B**) the observed standard deviations of coefficients **B **and *T*_(0.975,dg) _the two-sided *T*-student distribution value for a 95% confidence level and number of degrees of freedom (dg) equal to 1001 (observations) minus 47 (fluxes). All calculations were implemented by the authors in MATLAB (Version 7.0; Mathworks, USA).

### Hierarchical clustering

Groups of fluxes sharing similar strengths of association with productivity were hierarchically clustered on the two-dimensional space defined by the pair correlation coefficient/confidence interval. The Euclidian norm was chosen as distance measure. To evaluate if the classification presented in the dendrogram correlates well with the distance measurements between pairs of data objects, the cophenetic correlation coefficient (*c*) was calculated [49]. Values of *c *close to 1 indicate a good representation of the data. Additionally, for each link in the cluster tree, an inconsistency coefficient (*I*) was calculated, which compares the height of each link with that of neighbour links at the same level [50]. The higher the value of *I *is, the less similar the objects are. Thus, when plotting the number of emerging tree clusters against decreasing *I *values, a large discontinuity, along with the appearance of clusters with unacceptable small size, suggest a natural division in the dataset. All calculations were performed using predefined functions available in MATLAB (Statistics Toolbox, Multivariate Statistics section).

### Cell culture and virus handling

The host insect cell line *Sf*9 (ECACC 89070101) was maintained in 50 mL working volume shake flasks (Corning, USA) and kept in a humidified incubator operated at 27°C and 90 rpm. Sf900II serum- and protein-free medium (Gibco, Glasgow, UK) was used throughout this work. Cell density and viability were determined by cell counting using a Fuchs-Rosenthal chamber after diluting bulk samples in Trypan Blue.

The recombinant *Autographa californica *nucleopolyhedrovirus *Ac*-*vp39EGFP*, coding for the baculovirus major structural capsid protein, vp39, fused N-terminally to an EGFP reporter [51], was kindly provided by Dr. K. Airenne (University of Eastern Finland, Kuopio, Finland). Recombinant viruses were amplified by infecting *Sf9 *cells at 1x10^6 ^cells.mL^-1 ^with a MOI of 0.1 IP.cell^-1^, in 125 mL (working volume) spinner flasks (Wheaton, USA), and stored as culture supernatant at 4°C, protected from light. Virus titers were determined by an end-point dilution assay in 96-well plates, screening for GFP signal under an inverted fluorescence microscope [48].

### Infection experiments

Cells were cultured in 125 mL (working volume) spinner flasks or 50 mL working volume shake flasks (see Table 2). Infections with *Ac-vp39EGFP *were carried out at low (1x10^6 ^cells.mL^-1^) or high (3-4x10^6 ^cells.mL^-1^) cell density, using in all cases a low multiplicity of infection (0.1 IP.cell^-1^). Nutritional supplements were added at the time of infection as described in [27]. Briefly, concentrated stock solutions of sodium pyruvate, disodium α-ketoglutarate and L-glutamine (Sigma Aldrich, USA) were prepared in PBS and added to a final concentration of 12 mM, unless otherwise indicated. The amino acids mixture (Sigma Aldrich: R-7131) and complete Insect Medium Supplement (Sigma Aldrich: I-7267) were directly diluted 1:50 and 1:10 in culture medium, respectively, following indications of the manufacturer. In order to simulate nutrient limitations, cells were centrifuged prior to infection and ressuspended in a 1:1 dilution of conditioned medium with PBS buffer (adjusted to pH 6.1 and 370 mOsm). For AMPK activation, aminoimidazole carboxamide ribonucleotide (Sigma Aldrich: A-9978) was added to the culture at 6-8h post¬infection to a concentration of 500 μM [52,53].

### Metabolic profiling

Samples from infection experiments were collected at given time points and centrifuged at 1700xg for 10 minutes, at room temperature. Cell-free sterile supernatants were stored at 4°C for later virus titration, or at - 20°C to measure the concentration of sugars, lactate, ammonia, amino acids and carboxylates. Glucose and lactate concentrations were determined with automated enzymatic assays (YSI 7100 Multiparameter Bioanalytical System, USA). Ammonia was quantified enzymatically using a UV assay (No 1112732035; Boehringer Manheim, R-Biopharm AG, Germany). Maltose and sucrose were indirectly quantified after enzymatic hydrolysis using α-glucosidase and invertase from Sigma-Aldrich, respectively. Amino acid concentrations were profiled by high performance liquid chromatography (HPLC) using a reverse phase 3.9 × 150 mm column (AccQ.Tag, Waters, USA). A pre-column derivatization method (Waters AccQ.Tag Amino Acid Analysis) was used, as described in [27]. For the analysis of the carboxylic acids α-ketoglutarate and pyruvate, an ion-exclusion 8 × 300 mm sugar SH1011 column (Shodex, USA) was used [27].

## Authors' contributions

NC conceived the methodology, performed experimental cultures, analyzed the data and wrote the manuscript. VB participated in data acquisition, analysis and revised the manuscript. AT made substantial contribution to conception and revised the manuscript. MJTC and PMA contributed to the biological concept, revised the manuscript and gave final approval of the version to be published. RO was involved in the general design of the methodology, developed the *in silico *routines for data analysis and revised the manuscript.

## Supplementary Material

Additional file 1 **Metabolic reactions of the *Sf*9 cell line metabolism**. Includes a list of all stoichiometric reactions corresponding to exchange (measured) and intracellular (unknown) fluxes comprising the MFA model. References are provided for further information. Click here for file

Additional file 2 **Viral synthesis reactions used for complete MFA model establishment**. Includes information and references on the composition of insect viruses, and the set of viral synthesis reactions used to set the complete MFA model addressed in Table 1. Click here for file

Additional file 3 **Values of measured and MFA-estimated fluxes for all experiments**. Includes a table containing the values of all measured and MFA-estimated fluxes for the 13 experiments presented in this work. The later were used as direct inputs for correlation with a target vector of productivities (presented in Table 2) to establish hybrid MFA. Click here for file

## References

[B1] Lee SY Hong SH Lee DY Kim TY Yi-Ping Phoebe Chen Systems biotechnology: a new paradigm in biotechnology development Bioinformatics Technologies 2005 Springer Berlin Heidelberg 155 177 full_text

[B2] Otero JM Nielsen J Industrial Systems Biology Biotechnol Bioeng 2010 105 439 460 10.1002/bit.22592 19891008

[B3] Sauer U High-throughput phenomics: experimental methods for mapping fluxomes Curr Opin Biotechnol 2004 15 58 63 10.1016/j.copbio.2003.11.001 15102468

[B4] Gombert AK Nielsen J Mathematical modelling of metabolism Curr Opin Biotechnol 2000 11 180 186 10.1016/S0958-1669(00)00079-3 10753761

[B5] Kauffman KJ Prakash P Edwards JS Advances in flux balance analysis Curr Opin Biotechnol 2003 14 491 496 10.1016/j.copbio.2003.08.001 14580578

[B6] Burgard AP Pharkya P Maranas CD OptKnock: a bilevel programming framework for identifying gene knockout strategies for microbial strain optimization Biotechnol Bioeng 2003 84 647 657 10.1002/bit.10803 14595777

[B7] Pharkya P Burgard AP Maranas CD OptStrain: a computational framework for redesign of microbial production systems Genome Res 2004 14 2367 2376 10.1101/gr.2872004 15520298 PMC525696

[B8] Patil KR Rocha I Förster J Nielsen J Evolutionary programming as a platform for *in silico *metabolic engineering BMC Bioinformatics 2005 6 308 10.1186/1471-2105-6-308 16375763 PMC1327682

[B9] Pharkya P Maranas CD An optimization framework for identifying reaction activation/inhibition or elimination candidates for overproduction in microbial systems Metab Eng 2006 8 1 13 10.1016/j.ymben.2005.08.003 16199194

[B10] Fong SS Burgard AP Herring CD Knight EM Blattner FR Maranas CD Palsson BO In silico design and adaptive evolution of *Escherichia coli *for production of lactic acid Biotechnol Bioeng 2005 91 643 648 10.1002/bit.20542 15962337

[B11] Asadollahi MA Maury J Patil KR Schalk M Clark A Nielsen J Enhancing sesquiterpene production in *Saccharomyces cerevisiae *through *in silico *driven metabolic engineering Metab Eng 2009 11 328 334 10.1016/j.ymben.2009.07.001 19619667

[B12] Oberhardt MA Palsson BO Papin JA Application of genome-scale metabolic reconstructions Mol Syst Biol 2009 5 320 10.1038/msb.2009.77 19888215 PMC2795471

[B13] Covert MW Xiao N Chen TJ Karr JR Integrated metabolic, transcriptional regulatory and signal transduction models in Escherichia coli Bioinformatics 2008 24 2044 2050 10.1093/bioinformatics/btn352 18621757 PMC6702764

[B14] Lee JM Gianchandani EP Eddy JA Papin JA Dynamic analysis of integrated signalling, metabolic, and regulatory networks Plos Comput Biol 2008 4 e1000086 10.1371/journal.pcbi.1000086 18483615 PMC2377155

[B15] Kim J Reed JL OptORF: optimal metabolic and regulatory perturbations for metabolic engineering of microbial strains BMC Syst Biol 2010 4 53 10.1186/1752-0509-4-53 20426856 PMC2887412

[B16] Herrgard MJ Covert MW Palsson BO Reconstruction of microbial transcriptional regulatory networks Curr Opin Biotechnol 2004 15 70 77 10.1016/j.copbio.2003.11.002 15102470

[B17] Sidorenko Y Reichl U Structured model of influenza virus replication in MDCK cells Biotechnol Bioeng 2004 88 1 14 10.1002/bit.20096 15384040

[B18] Boghigian BA Seth G Kiss R Pfeifer BA Metabolic flux analysis and pharmaceutical production Metab Eng 2010 12 81 95 10.1016/j.ymben.2009.10.004 19861167

[B19] Quek LE Dietmair S Krömer JO Nielsen LK Metabolic flux analysis of mammalian cell culture Metab Eng 2010 12 161 171 10.1016/j.ymben.2009.09.002 19833223

[B20] Sheikh K Förster J Nielsen LK Modeling hybridoma cell metabolism using a generic genome-scale metabolic model of *Mus musculus* Biotechnol Prog 2005 21 112 121 10.1021/bp0498138 15903248

[B21] Wold S Sjöström M Eriksson L PLS-regression: a basic tool of chemometrics Chemometr Intell Lab 2001 58 109 130 10.1016/S0169-7439(01)00155-1

[B22] Ikonomou L Schneider YJ Agathos SN Insect cell culture for industrial production of recombinant proteins Appl Microbiol Biotechnol 2003 62 1 20 10.1007/s00253-003-1223-9 12733003

[B23] Summers MD Milestones leading to the genetic engineering of baculoviruses as expression vector systems and viral pesticides Adv Virus Res 2006 68 3 73 10.1016/S0065-3527(06)68001-9 16997008

[B24] Kost TA Condreay JP Recombinant baculoviruses as mammalian cell gene-delivery vectors Trends Biotechnol 2002 20 173 180 10.1016/S0167-7799(01)01911-4 11906750

[B25] Bernal V Carinhas N Yokomizo AY Carrondo MJT Alves PM Cell density effect in the baculovirus-insect cells system: a quantitative analysis of energetic metabolism Biotechnol Bioeng 2009 104 162 180 10.1002/bit.22364 19459142

[B26] Carinhas N Bernal V Yokomizo AY Carrondo MJT Oliveira R Alves PM Baculovirus production for gene therapy: the role of cell density, multiplicity of infection and medium exchange Appl Microbiol Biotechnol 2009 81 1041 1049 10.1007/s00253-008-1727-4 18923829

[B27] Carinhas N Bernal V Monteiro F Carrondo MJT Oliveira R Alves PM Improving baculovirus production at high cell density through manipulation of energy metabolism Metab Eng 2010 12 39 52 10.1016/j.ymben.2009.08.008 19732849

[B28] Burnham AJ MacGregor JF Viveros R Interpretation of regression coefficients under a latent variable regression model J Chemometr 2001 15 265 284 10.1002/cem.680

[B29] Menze MA Clavenna MJ Hand SC Depression of cell metabolism and proliferation by membrane-permeable and -impermeable modulators: role for AMP-to-ATP ratio Am J Physiol Regul Integr Comp Physiol 2005 288 501 510 10.1152/ajpregu.00490.2004 15458972

[B30] Boulesteix AL Strimmer K Partial least squares: a versatile tool for the analysis of high-dimensional genomic data Brief Bioinform 2006 8 32 44 10.1093/bib/bbl016 16772269

[B31] Teixeira AP Carinhas N Dias JML Cruz P Alves PM Carrondo MJT Oliveira R Hybrid semi-parametric mathematical systems: bridging the gap between systems biology and process engineering J Biotechnol 2007 132 418 425 10.1016/j.jbiotec.2007.08.020 17870200

[B32] Lee KH Park JH Kim TY Kim HU Lee SY Systems metabolic engineering of *Escherichia coli *for L-threonine production Mol Syst Biol 2007 3 149 10.1038/msb4100196 18059444 PMC2174629

[B33] Zelle RM de Hulster E van Winden WA de Waard P Dijkema C Winkler AA Geertman JM van Dijken JP Pronk JT van Maris AJ Malic acid production by *Saccharomyces cerevisiae*: engineering of pyruvate carboxylation, oxaloacetate reduction, and malate export Appl Environ Microbiol 2008 74 2766 2777 10.1128/AEM.02591-07 18344340 PMC2394876

[B34] Sauer U Metabolic networks in motion: ^13^C-based flux analysis Mol Syst Biol 2006 2 62 10.1038/msb4100109 17102807 PMC1682028

[B35] O'Callaghan PM James DC Systems biotechnology of mammalian cell factories Brief Funct Genomic Proteomic 2008 7 95 110 18326543 10.1093/bfgp/eln012

[B36] Ma HU Zhao XM Yuan YJ Zeng AP Decomposition of metabolic network into functional modules based on the global connectivity structure of reaction graph Bioinformatics 2004 20 1870 1876 10.1093/bioinformatics/bth167 15037506

[B37] Guimerà R Amaral LAN Functional cartography of complex metabolic networks Nature 2005 433 895 900 15729348 10.1038/nature03288 PMC2175124

[B38] Schuster S Fell DA Dandekar T A general definition of metabolic pathways useful for systematic organization and analysis of complex metabolic networks Nature Biotechnol 2000 18 326 332 10.1038/73786 10700151

[B39] Papin JA Stelling J Price ND Klamt S Schuster S Palsson BO Comparison of network-based pathway analysis methods Trends Biotechnol 2004 22 400 405 10.1016/j.tibtech.2004.06.010 15283984

[B40] Melzer G Esfandabadi ME Franco-Lara E Wittmann C Flux Design: In silico design of cell factories based on correlation of pathway fluxes to desired properties BMC Syst Biol 2009 3 120 10.1186/1752-0509-3-120 20035624 PMC2808316

[B41] Blazeck J Alper H Systems metabolic engineering: genome-scale models and beyond Biotechnol J in press 20151446 10.1002/biot.200900247 PMC2911524

[B42] Lee JM Gianchandani EP Papin JA Flux balance analysis in the era of metabolomics Brief Bioinform 2006 7 140 150 10.1093/bib/bbl007 16772264

[B43] Stephanopoulos G Aristidou AA Nielsen J Metabolic engineering. Principles and Methodologies 1998 San Diego: Academic Press

[B44] Wang NS Stephanopoulos G Application of macroscopic balances to the identification of gross measurement errors Biotechnol Bioeng 1983 25 2177 2208 10.1002/bit.260250906 18574815

[B45] Klamt S Stelling J Ginkel M Gilles ED FluxAnalyzer: exploring structure, pathways, and flux distributions in metabolic networks on interactive flux maps Bioinformatics 2003 19 2 10.1093/bioinformatics/19.2.261 12538248

[B46] Andersson CA Bro R The N-way Toolbox for MATLAB Chemom Intell Lab Syst 2000 52 1 4 10.1016/S0169-7439(00)00071-X

[B47] Jong S SIMPLS: An Alternative Approach to Partial Least Squares Regression Chemometr Intell Lab 1993 18 251 263 10.1016/0169-7439(93)85002-X

[B48] Roldão A Oliveira R Carrondo MJT Alves PM Error assessment in recombinant baculovirus titration: evaluation of different methods J Virol Methods 2009 159 69 80 19442848 10.1016/j.jviromet.2009.03.007

[B49] Sokal RR Rohlf FJ The comparison of dendrograms by objective methods Taxon 1962 11 33 40 10.2307/1217208

[B50] Cordes D Haughton V Carew JD Arfanakis K Maravilla K Hierarchical clustering to measure connectivity in fMRI resting-state data Magn Reson Imaging 2002 20 305 317 10.1016/S0730-725X(02)00503-9 12165349

[B51] Kukkonen SP Airenne KJ Marjomaki V Laitinen OH Lehtolainen P Kankaanpaa P Mahonen AJ Raty JK Nordlund HR Oker-Blom C Kulomaa MS Yla-Herttuala S Baculovirus capsid display: a novel tool for transduction imaging Mol Ther 2003 8 853 862 10.1016/j.ymthe.2003.07.009 14599820

[B52] Xiao W Yang Y Weng Q Lin T Yuan M Yang K Pang Y The role of the PI3K-Akt signal transduction pathway in *Autographa californica *multiple nucleopolyhedrosisvirus infection of *Spodoptera frugiperda *cells Virology 2009 391 83 89 10.1016/j.virol.2009.06.007 19573890

[B53] Sullivan JE Brocklehurst KJ Marley AE Carey F Carling D Beri RK Inhibition of lipolysis and lipogenesis in isolated rat adypocytes with AICAR, a cell-permeable activator of AMP-activated protein kinase FEBS Lett 1994 353 33 36 10.1016/0014-5793(94)01006-4 7926017

