# Supplementary material for: Hybrid metabolic flux analysis: combining stoichiometric and statistical constraints to model the formation of complex recombinant products
Source: BMC Syst Biol. 2011 Feb 25;5:34. doi: 10.1186/1752-0509-5-34 (PMC3236310; doi:10.1186/1752-0509-5-34)
Supplement: Additional file 1 — Metabolic reactions of the Sf9 cell line metabolism. Includes a list of all stoichiometric reactions corresponding to exchange (measured) and intracellular (unknown) fluxes comprising the MFA model. References are provided for further information. [file 1752-0509-5-34-S1.PDF]

ADDITIONAL FILE 1: Metabolic reactions of the *Spodoptera frugiperda* cell line (*Sf*9) metabolism.

For a description of the following, see Bernal et al. (2009) and Carinhas et al. (2010).

The following measured exchange fluxes were considered as constraints for MFA calculations.

Transporters/passive diffusion

Glc_e = Glc

$Malt_e$ = Malt

$Sucr_e$ = Sucr

Lac = Lac_e

Amm = Amm_e

Pyr_e = Pyr

αKG_e = αKG

Amino acids Transport

Glu_e + 1 ATP = Glu + 1 ADP

Asp_e + 1 ATP = Asp + 1 ADP

Gln_e + 0.33 ATP = Gln + 0.33 ADP

Ser_e + 0.33 ATP = Ser + 0.33 ADP

Gly_e + 0.33 ATP = Gly + 0.33 ADP

Thr_e + 0.33 ATP = Thr + 0.33 ADP

Met_e + 0.33 ATP = Met + 0.33 ADP

Pro_e + 0.33 ATP = Pro + 0.33 ADP

Asn_e + 0.33 ATP = Asn + 0.33 ADP

Leu_e + 0.33 ATP = Leu + 0.33 ADP

Ile_e + 0.33 ATP = Ile + 0.33 ADP

Val_e + 0.33 ATP = Val + 0.33 ADP

Phe_e + 0.33 ATP = Phe + 0.33 ADP

Tyr_e + 0.33 ATP = Tyr + 0.33 ADP

His_e + 0.33 ATP = His + 0.33 ADP

Lys_e + 0.33 ATP = Lys + 0.33 ADP

Arg_e + 0.33 ATP = Arg + 0.33 ADP

Ala = Ala_e

Biomass formation

μ

Metabolic reactions of *S. frugiperda*'s metabolism estimated by MFA and used for PLS modelling.

Glycolysis and pentose-phosphate pathway (PPP)

Glc + ATP = G6P + ADP (*hexokinase*)

Malt = Glc + G6P (set equal to $Malt_e$ = Malt)

Sucr + 2 ATP = G6P + F6P (set equal to $Sucr_e$ = Sucr)

G6P = F6P *(phospho-gluco isomerase)*

F6P + ATP = 2 GAP

GAP + NAD^+ + ADP = NADH + ATP + PEP

ADP + PEP = Pyr + ATP (*pyruvate kinase*)

3 G6P + 6 $NADP^+$ = 3 CO_2 + 3 R5P + 6 NADPH (*pentose-phosphate pathway*)

Pyruvate node

Pyr + NADH = Lac + NAD^+ (*lactate dehydrogenase,* set equal to Lac = Lac_e)

Pyr + Glu = Ala + αKG (*alanine aminotranferase*)

Pyr + NAD^+ = CO_2 + ACoA + NADH (*pyruvate dehydrogenase*)

Mal = Pyr + CO_2 (*malic enzyme*)

TCA cycle

ACoA + OAA = Cit (*citrate synthase*)

Cit + NAD^+ = CO_2 + αKG + NADH

αKG + NAD$^+$ = CO_2 + SuCoA + NADH *(αKG dehydrogenase)*

SuCoA + ADP = Suc + ATP (*succinyl coenzyme A synthetase*)

Suc + FAD = Fum + $FADH_2$ (*succinate dehydrogenase*)

Fum = Mal (*fumarase*)

Mal + NAD$^+$ = OAA + NADH (*malate dehydrogense*)

Amino acids metabolism

αKG + Gln + NADH = 2 Glu + NAD$^+$ (*glutamate synthase*)

Glu + NAD$^+$ = αKG + Amm + NADH (*glutamate dehydrogenase*)

2 Gly + NAD$^+$ = CO_2 + Amm + Ser + NADH

Ser = Pyr + Amm

2 αKG + Lys + 4 NAD$^+$ + NADPH + FAD = 2 CO_2 + 2 Glu + 2 ACoA + 4 NADH + NADP + $FADH_2$

αKG + Leu + NAD$^+$ + ATP + FAD = CO_2 + Glu + 2 ACoA + NADH + ADP + $FADH_2$ + mevalonate

αKG + Ile + 2 NAD$^+$ + ATP + FAD = CO_2 + Glu + ACoA + SuCoA + 2 NADH + ADP + $FADH_2$

αKG + Arg + NAD$^+$ = 2 Glu + NADH + urea

Glu + OAA = αKG + Asp (*aspartate aminotransferase*)

Thr + NAD$^+$ = ACoA + Gly + NADH

αKG + Val + 3 NAD$^+$ + ATP + FAD = CO_2 + Glu + SuCoA + 3 NADH + ADP + $FADH_2$

Ser + Met + NAD$^+$ + ATP = SuCoA + Amm + Cys + NADH + 2 ADP

Phe + NADH + O_2 = Tyr + NAD$^+$

αKG + Tyr + O_2 = CO_2 + Glu + ACoA + Fum

His = Glu + Amm

Asn = Amm + Asp

Pro + NADP$^+$ + 0.5 O_2 = Glu + NADPH

Respiration fluxes

NADH + 2.5 ADP + 0.5 O_2 = NAD$^+$ + 2.5 ATP

1.5 ADP + $FADH_2$ + 0.5 O_2 = 2.5 ATP + FAD

ATP = Energy

$O_{2,e} = O_2$

$CO_2 = CO_{2,e}$

Transhydrogenase

NAD^+ + NADPH = NADH + $NADP^+$

Biomass synthesis

0.5 CO_2 + R5P + 1.9 Gln + 1.3 Ser + 1.3 Asp + 0.7 NAD^+ + NADPH + 9 ATP = 1.9 Glu + 0.8 Fum + 0.8 Gly + DNA + 0.7 NADH + $NADP^+$ + 9 ADP

CO_2 + R5P + 1.9 Gln + Ser + 1.3 Asp + NAD^+ + ATP = Glu + 0.8 Fum + 0.5 Gly + RNA + NADH + ADP

0.417 Ala + 0.251 Glu + 0.315 Gln + 0.432 Gly + 0.348 Ser + 0.344 Lys + 0.410 Leu + 0.213 Ile + 0.301 Arg + 0.238 Asp + 0.290 Thr + 0.305 Val + 0.109 Met + 0.162 Phe + 0.131 Tyr + 0.109 His + 0.266 Pro + 0.223 Asn + 0.142 Cys + 10.014 ATP = 1 Protein + 10.014 ADP

9 AcoA + 7 NADPH + 26 ATP + 10 NADH + 1 O_2 = 1 FA + 7 $NADP^+$ + 26 ADP + 10 NAD^+

1000 Protein + 111 FA + 89.5 DNA + 224.7 RNA = Biomass

(The balances of ADP, urea, mevalonate and "Energy" could not be closed, as these metabolites intervene in biochemical processes not considered in the metabolic network.)

References

Bernal V, Carinhas N, Yokomizo AY, Carrondo MJT, Alves PM: **Cell density effect in the Baculovirus-Insect Cells system: a quantitative analysis of energetic metabolism.** *Biotechnol Bioeng* 2009, **104**:162-180.

Carinhas N, Bernal V, Monteiro F, Carrondo MJT, Oliveira R, Alves PM: **Improving baculovirus production at high cell density through manipulation of energy metabolism.** *Metab Eng* 2010, **12**:39-52.
