# Supplementary material for: Hybrid metabolic flux analysis: combining stoichiometric and statistical constraints to model the formation of complex recombinant products
Source: BMC Syst Biol. 2011 Feb 25;5:34. doi: 10.1186/1752-0509-5-34 (PMC3236310; doi:10.1186/1752-0509-5-34)
Supplement: Additional file 2 — Viral synthesis reactions used for complete MFA model establishment. Includes information and references on the composition of insect viruses, and the set of viral synthesis reactions used to set the complete MFA model addressed in Table 1. [file 1752-0509-5-34-S2.PDF]

ADDITIONAL FILE 2: Viral synthesis reactions used for complete MFA model establishment.

Insect baculoviruses were considered to be composed of 77% protein and 8% DNA (Bergold and Wellington, 1954). Molar percentages of amino acids in insect viruses were taken as the average values from Wellington (1954). Each viral particle was considered to weight 1.07×10^{-17} g and has a circular genome of 262000 deoxyribonucleotides.

Viral protein synthesis

0.00305 Ala + 0.00371 Glu + 0.00231 Gln + 0.00388 Gly + 0.00703 Ser + 0.00226 Lys + 0.00513 Leu + 0.00374 Ile + 0.01229 Arg + 0.00603 Asp + 0.00449 Thr + 0.0028 Val + 0.00167 Met + 0.0037 Phe + 0.00385 Tyr + 0.00095 His + 0.00431 Pro + 0.00336 Asn + 0.00057 Cys = 1 bacProtein

(1 bacProtein represents the pmol content of protein amino acids present in 10^6 particles; ATP utilization was not taken into account.)

Viral DNA synthesis

0.5 CO_2 + R5P + 1.9 Gln + 1.3 Ser + 1.3 Asp + 0.7 NAD + NADPH + 9 ATP = 1.9 Glu + 0.8 Fum + 0.8 Gly + bacDNA + 0.7 NADH + NADP + 9 ADP

(1 DNA represents 1 “average” deoxyribonucleotide, considering a G/C content of 40% (The International Lepidopteran Genome Project).)

Lumped reaction

0.000435 bacDNA + 0.001 bacProtein = 1 bacMass

(1 bacMass represents 10^6 particles; the coefficients represent the nmol content of each component in 10^6 particles.)

Measured outflux of viral particles

vBac

References

Bergold GH, Wellington EF: **Isolation and chemical composition of the membranes of an insect virus and their relation to the virus and polyhedral bodies.** *J Bacteriol* 1954, **67**:210-216.

Wellington EF: **The amino acid composition of some insect viruses and their characteristic inclusion-body proteins.** *Biochem J* 1954, **57**:334-338.
